# A Roadmap for Building Waterborne Virus Traps

**DOI:** 10.1021/jacsau.2c00377

**Published:** 2022-10-04

**Authors:** Antonius Armanious, Raffaele Mezzenga

**Affiliations:** † Department of Health Sciences and Technology, ETH Zurich, Zurich8092, Switzerland; ‡ Department of Materials, ETH Zurich, Zurich8093, Switzerland

**Keywords:** waterborne viruses, adsorption-based filtration, viruses at solid−water interfaces, physicochemical properties of viruses, point-of-use water treatment, multi-adsorbate systems, virus inactivation at interfaces, virus traps

## Abstract

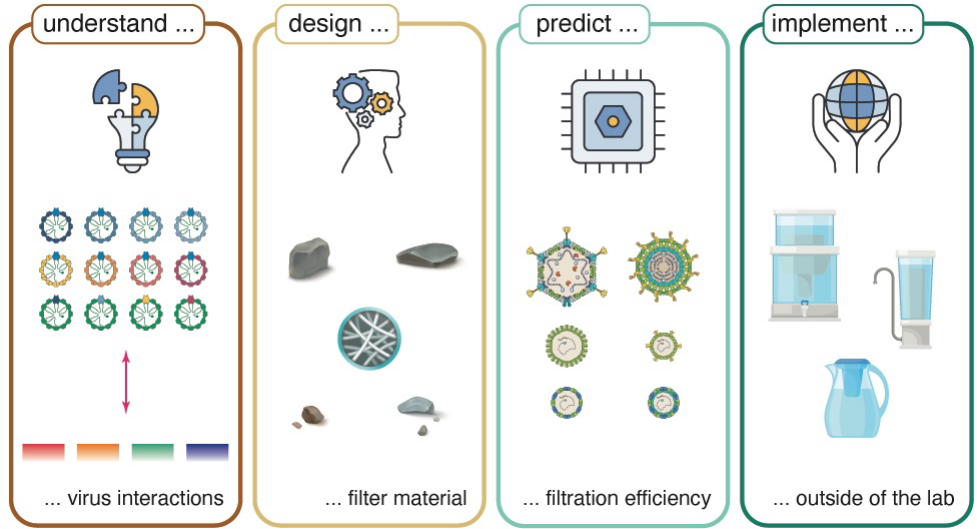

Outbreaks of waterborne viruses pose a massive threat to human health, claiming the lives of hundreds of thousands of people every year. Adsorption-based filtration offers a promising facile and environmentally friendly approach to help provide safe drinking water to a world population of almost 8 billion people, particularly in communities that lack the infrastructure for large-scale facilities. The search for a material that can effectively trap viruses has been mainly driven by a top-down approach, in which old and new materials have been tested for this purpose. Despite substantial advances, finding a material that achieves this crucial goal and meets all associated challenges remains elusive. We suggest that the road forward should strongly rely on a complementary bottom-up approach based on our fundamental understanding of virus interactions at interfaces. We review the state-of-the-art physicochemical knowledge of the forces that drive the adsorption of viruses at solid–water interfaces. Compared to other nanometric colloids, viruses have heterogeneous surface chemistry and diverse morphologies. We advocate that advancing our understanding of virus interactions would require describing their physicochemical properties using novel descriptors that reflect their heterogeneity and diversity. Several other related topics are also addressed, including the effect of coadsorbates on virus adsorption, virus inactivation at interfaces, and experimental considerations to ensure well-grounded research results. We finally conclude with selected examples of materials that made notable advances in the field.

## Introduction

Contaminated drinking water is responsible for more than 500 000 deaths annually, mostly among children under 5 years old.^1^ Waterborne enteric viruses, e.g., Enteroviruses, Adenoviruses, and Rotaviruses, can cause diseases, such as diarrhea and dysentery, with approximately 40% of often-fatal childhood diarrhea in developing countries being caused by viral infections,^2^ not to mention the hospitalization and massive socioeconomic costs.^3,4^ While most waterborne virus outbreaks occur in less privileged communities,^1−5^ communities with state-of-the-art wastewater and water treatment facilities are still prone to waterborne virus outbreaks.^6−14^ Waterborne viruses are mainly transmitted through the fecal-oral route, which can primarily be interrupted through effective wastewater and water treatment approaches. Currently available approaches are either of nonsufficient efficacy, come at high environmental and economic costs, or require advanced infrastructure, and thus are not accessible to large portions of the world population. Novel forward-looking approaches are required to meet the urgent need of providing safe drinking water to a world population of almost 8 billion people, while simultaneously protecting the environment from hazardous chemicals and greenhouse gas emissions.

Traditionally, disinfection, coagulation, and ultrafiltration are the most commonly used approaches for water purification from viruses. Disinfection is typically conducted using chemical disinfectants or UV light. While regarded as one of the most effective approaches against viruses, disinfection still shows varying efficiencies, even among closely related viruses with very subtle genetic and structural differences.^15−17^ Additionally, disinfection byproducts, especially when using chlorine-based disinfectants in the presence of natural organic matter (NOM), are toxic to the environment and humans.^18,19^ Consequently, it is necessary to completely purify the water from NOMs to avoid the production of these toxic byproducts, which in itself constitutes a major challenge. Moreover, the efficacy of disinfection approaches is largely compromised by the aggregation of viruses.^20−24^ Both chemical and UV disinfection come at high operational costs and expertise, making their accessibility limited to a small portion of the global population. Coagulation is driven by adding a chemical, i.e., the coagulant, that causes colloidal instability, forming larger particles that can sediment faster. While being a relatively simple process that can be utilized against a broad spectrum of colloids, its efficacy against viruses is considerably compromised due to both the abundance of other colloids, such as NOM, and variations in water chemistry and composition.^25,26^ The need to continuously feed the system with coagulants, usually aluminum or iron salts, comes at a relatively high cost and is thus also inaccessible to a large portion of the world population. Filtration by physical size exclusion is only possible using ultrafiltration or nanofiltration with pore sizes smaller than 20 nm.^27^ These filters require high overhead pressure, periodic back flushing, and chemical cleaning, which all impose markedly high environmental and financial costs.^28,29^ In the cases of coagulation and filtration, viruses are usually still infectious after treatment; they are retained in the form of sediment or filter retentate, which, if not properly treated, could pose a higher risk.^30^ Finally, none of these approaches is suitable for point-of-use (POU) application in developing communities. POU is the most promising water purification strategy in susceptible communities which lack the requisite infrastructure and expertise for large-scale water and wastewater treatment facilities, or where contamination occurs in the so-called last km, i.e., in the water distribution systems shortly before it reaches the consumer.

Adsorption-based filtration of viruses has emerged as an alternative, with the potential to overcome the limitations of the traditionally used purification methods. Adsorption-based filtration is in particular characterized by the low energy and financial cost of operation, the lack of use of chemicals, the facile operational expertise, and the variety of materials that could be used for building such filters. However, for it to achieve the needed global impact in fighting virus dissemination, adsorption-based filtration must fulfill many criteria: environmentally friendly, low cost, and wide availability; chemical and mechanical stability; high adsorption capacity and efficiency; and simple regeneration and reuse.^31^ Particularly important is that such filters need to inactivate the adsorbed viruses, i.e., render them noninfectious, either directly upon adsorption or during the regeneration processes.

Fulfilling these criteria is a major challenge, partly due to the several physical and biological processes^32,33^ that could hinder bringing the viruses in close proximity to the adsorbing filter material, but primarily due to the lack of a material that can efficiently trap viruses. The latter is mainly attributed to the following reasons: (i) viruses cover a broad range of physicochemical properties; a material that would adsorb one virus might not adsorb the other; (ii) there is an abundance of other contaminants in water and wastewater, particularly NOM, which often compromises the efficacy of virus adsorption and retention; and (iii) the chemical properties of water (i.e., pH, ionic strength, ionic composition) and the concentration and type of other contaminants (e.g., NOM, heavy metals, and organic compounds) are continuously changing, which may consequently affect the efficacy of the clarification process.

A large body of research over the last two decades has been driven by a top-down approach, in which various old and novel materials have been tested to adsorb viruses. These activities revealed several essential material properties that are necessary for high efficacy filtration. Still, the search for a material that fulfills the aforementioned criteria remains elusive, resulting in very rare examples that made it to real-life applications outside of the laboratory. In this work, we advocate that, for adsorption-based filtration to achieve its goal, it needs to be complemented by a bottom-up approach. Specifically, this approach must start with a fundamental understanding of the interactions that drive virus adsorption to solid–water interfaces, and how this depends on the physicochemical properties of viruses and the adsorbing surface. Such knowledge would dictate the designing principles for new materials to overcome the limitations of existing materials.

This Perspective starts by providing a short introduction to waterborne viruses and how to address the challenge of extrapolating experimental results to the broad range of waterborne viruses. It then presents a state-of-the-art understanding of virus interactions at solid–water interfaces, followed by a discussion on the existing knowledge gaps and how to address them. Afterward, a brief discussion is presented on the potential mechanisms of virus inactivation at solid–water interfaces and what could still be explored to design a material that not only traps but also inactivates viruses. A few essential experimental considerations to ensure nonambiguous interpretation of results for future studies are also identified. Finally, we conclude with selected examples of materials used for adsorption-based filtration of viruses that made notable advances in the field.

## Waterborne Viruses, Their Surrogates, and the Future of Virus Research

**Figure 1:**
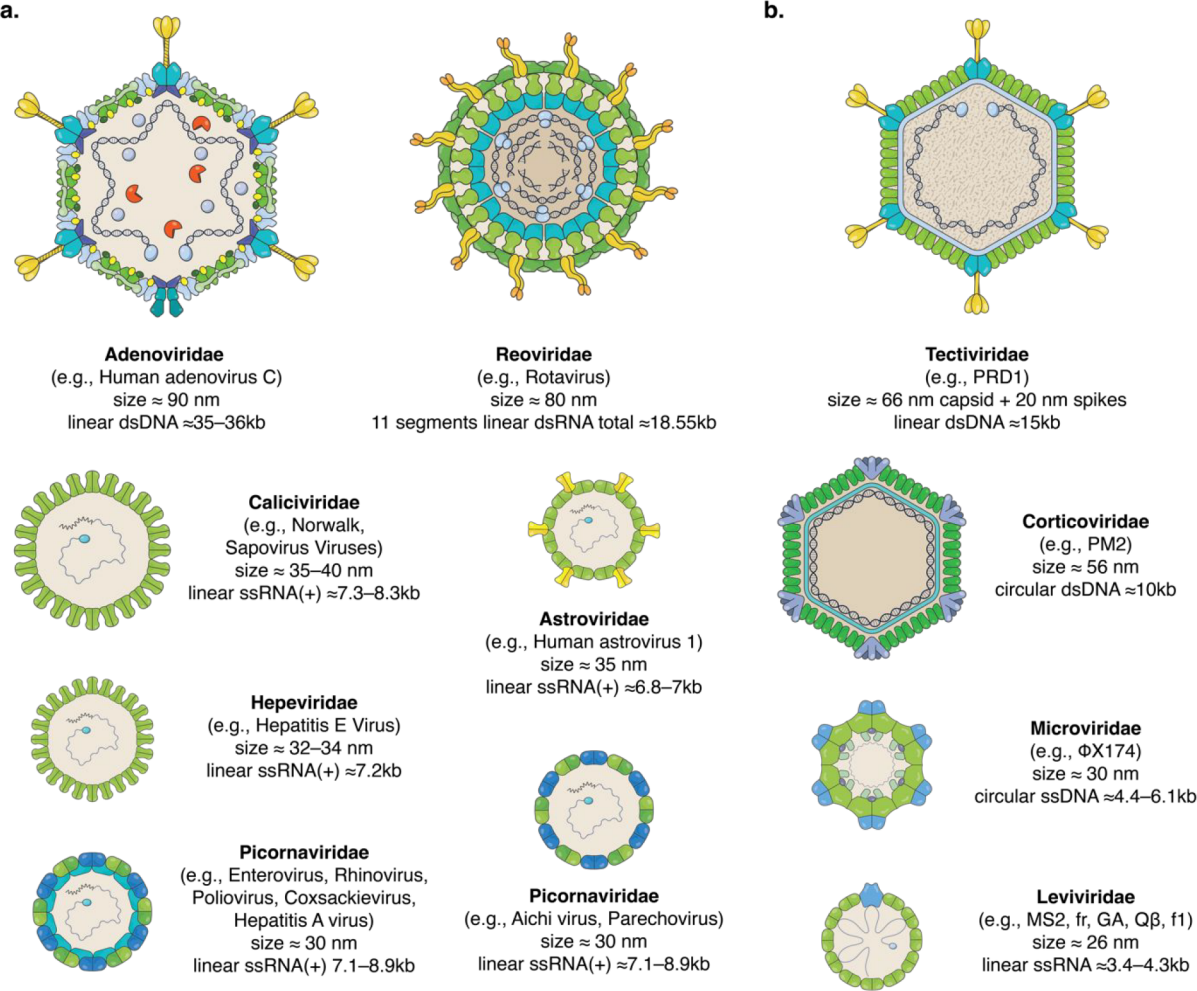
Illustrations of selected waterborne human viruses and bacteriophages. Representations of six different families of human viruses (a) and four families of bacteriophages (b). The illustrations highlight the very broad diversity of virus structure, size, and genome type and size. Virus illustrations are reproduced with permission;^168^ the illustrations are nonidentical reproductions of the original pictures. Data about sizes, genome type, and length of genomes were obtained from viralzone.expasy.org.^169^

Viruses are infectious agents that use the cellular machinery of their host, e.g., humans, animals, or bacteria, to make replicas of themselves. Viruses can be classified according to different criteria, including genome type, host, and structure. Here, we are particularly concerned with human waterborne viruses, which are mostly nonenveloped; i.e., they do not contain a lipid membrane around the proteinaceous capsid and the encapsulated genome. The lack of an envelope contributes to their robustness, retaining their viability for extended periods of time even under harsh environmental conditions outside of their host.^34,35^Figure 1a shows illustrative representations of some of the most common waterborne viruses: human Adenovirus, Astrovirus, Norwalk virus (Norovirus), Sapovirus, Hepatitis E virus, Enterovirus, Rhinovirus, Poliovirus, Coxsackievirus, Hepatitis A virus, Aichi virus, Parechovirus, and Rotavirus; the illustrations highlight the vast diversity of these viruses. These viruses relate to a multitude of diseases, including gastroenteritis, respiratory diseases, conjunctivitis, cystitis, hepatitis, paralysis, meningitis, hand-foot-and-mouth disease, heart anomalies, skin rash, and encephalitis.^36^

Conducting studies with human viruses is not free from biosafety risks and is also very costly and time-consuming. In vitro propagation of human viruses to sufficiently high concentrations, relevant for filtration studies, is in many cases not attainable within reasonable time frames and costs. Some human viruses are not even culturable in vitro with no available infectivity assays, e.g., Noroviruses.^37−39^ For these reasons, and with only few exceptions, most virus filtration studies are conducted using bacteriophages,^40,41^ i.e., viruses that infect bacteria. Most bacteriophages pose no health risks to humans, they can be propagated to very high concentrations, and their infectivity can often be readily assessed within less than 24 h; culturable human viruses, on the other hand, require between 2–14 days to assess their infectivity.^42,43^Figure 1b shows some of the well-studied bacteriophages, among which MS2 and other viruses from the *Leviviridae* family are the most commonly used for filtration/inactivation research.^40,41^ The advantages offered by using bacteriophages must be considered with a caveat in mind: knowledge obtained using one virus is not directly transferable to another virus; this does not only apply to knowledge transfer from bacteriophages to human viruses, but also among bacteriophages and human viruses themselves. Several studies have demonstrated that even small differences between viruses, often from the same family, exhibit striking differences in their inactivation kinetics,^16,17^ interactions at interfaces,^44,45^ and purification efficiencies.^46,47^ Therefore, obtaining meaningful results without having to run virus filtration/inactivation studies using all relevant human viruses, which is literally impossible, remains a challenge.

There are two options to address this challenge. The first is to use reference organisms. For example, the World Health Organization (WHO) recommends the use of Rotavirus as a reference organism for waterborne viruses.^48^ While sometimes otherwise claimed, such reference organisms often do not represent the worst-case scenario and are usually chosen based on the availability and simplicity of propagation and quantification methods. Taking the diversity of waterborne viruses and the variety of filtration/inactivation mechanisms into account, it is improbable to find one virus that represents the worst-case scenario for all of these processes. Therefore, using reference organisms might result in overestimating the filtration/inactivation efficiencies with adverse consequences when the tested technologies are used out of the laboratory context. The other viable option is to study an array of viruses/bacteriophages to gain a mechanistic understanding of virus filtration/inactivation and correlating these mechanisms to the physicochemical properties of the viruses. In this way, one can use the physicochemical properties of any other virus to predict its filtration/inactivation efficiency. Indeed, several research groups have followed this approach over the past decade.^15,16,44,45,49−51^ However, and despite the advances achieved in offering a mechanistic understanding of various virus adsorption and inactivation processes, the predictive power is still lacking.

To better identify the key requirements for achieving this predictive power, we take inspiration from predicting the environmental fate of small organic molecules. For example, the development of the *n*-octanol/water partition coefficient, *k*_ow_,^52^ for organic molecules has been successfully used to predict their environmental fate, e.g., bioaccumulation/bioconcentration,^53,54^ water solubility,^55,56^ soil/sediment attachment coefficients,^57^ and distribution in different cellular compartments.^58,59^ This was only possible due to the availability of experimentally determined *k*_ow_ for tens of organic molecules as well as data about their environmental fate. These data enabled the development and validation of computational models that can estimate *k*_ow_ for new molecules, which is particularly useful for organic molecules that are challenging to investigate experimentally. Moreover, based on these data, it was possible to establish and verify predictive models that can anticipate the environmental fate of experimentally challenging and/or novel organic molecules. The possibility to take most of the work from the laboratory bench to the computer enables rapid and cost-effective screening of a large number of molecules with a fraction of the cost and time required if this work was to be done experimentally. It is also important to note that, for some organic molecules, experimental assessment is practically impossible.

The example of the *n*-octanol/water partition coefficient highlights the key hurdle to developing predictive models for viruses, i.e., the limited number of experimentally accessible viruses, which impedes the development and validation of broadly applicable models. With only few exceptions,^16,44,45,47,60,61^ most virus studies are conducted using one to three viruses. These viruses are either selected to be as diverse as possible, e.g., different genome type, size, and morphology,^47,60^ or as similar as possible with systematic variation in their physicochemical properties.^16,44,45^ The former offers an overview of the diversity in virus interactions but can hardly yield any mechanistic understanding. The latter, due to the similarity of the chosen viruses, could unintentionally result in a biased mechanistic understanding of virus interactions.^62,63^ Additionally, even carefully selected viruses with systematic variation in their physicochemical properties still exhibit variations in multiple properties simultaneously, e.g., charge and hydrophobicity.^16,44,45^ Selective variation of one property, e.g., charge, while maintaining all other properties fixed is a prerequisite for attaining a detailed quantitative understanding of virus interactions. Unlike synthesized organic molecules, naturally existing viruses do not offer such versatility. Genetic engineering of viruses, particularly bacteriophages, has long been a difficult and labor-intensive process,^64,65^ hindering the production of bacteriophages with tailored properties for virus studies. Recent advances in bacteriophage engineering streamlined this process, enabling targeted gene editing of bacteriophages within less than a week.^64−67^ We envision future virus studies to be conducted using an array of genetically engineered bacteriophages to pinpoint the key mechanisms of interactions, as well as build predictive models that could be used to anticipate the interactions of human viruses.

The design of genetically engineered bacteriophages has to be guided by the aim of understanding how virus composition and structure relate to virus adsorption and inactivation. In the following section, we discuss selected virus interactions that are crucial for virus adsorption and inactivation at interfaces. We pay particular attention to knowledge gaps and how such gaps could potentially be filled by utilizing engineered bacteriophages and/or other approaches.

## Virus Interactions at Solid–Water Interfaces

Viruses are not motile; i.e., they are not capable of actively moving by themselves. Their mobility outside and inside of their host is solely driven by external forces and their surface interactions. From a physical chemistry point of view, they can be considered as abiotic colloids. A large body of literature suggests that virus interactions to solid–water interfaces are primarily driven by electrostatic forces^44−46,68−88^ and the hydrophobic effect.^44,69,75,88−95^ Many of the adsorption-based filters intentionally or nonintentionally exploit these two interactions to drive the adsorption of viruses. Most waterborne viruses are thought to carry a net negative charge at circumneutral pH values.^96^ It is, therefore, logical to use positively charged material to trap the negatively charged viruses. While this approach has shown promising results,^46,70,71,73,74^ noticeable variation in efficacy across different viruses has been observed,^46,73,79^ raising doubts about its efficacy against the broad range of waterborne viruses and under varying water chemistry. In addition, both positively charged and hydrophobic surfaces are susceptible to competitive adsorption from other adsorbates, particularly NOM.^45,97^ More innovative approaches are needed in order to circumvent these challenges. For instance, it has been shown that some viruses still adsorb to negatively charged surfaces under weak electrostatic repulsive conditions,^44^ while no detectable adsorption for NOMs was observed under the same conditions.^97^ These results point to a potential approach for developing filtration membranes that are repulsive to competitors, e.g., NOM, while still attractive to viruses. However, successful development of such a membrane or other ones would only be possible through a clear fundamental understanding of the effects of electrostatic interactions, the hydrophobic effect, water chemistry, and other adsorbates on virus attachment at solid–water interfaces. As previously mentioned, such understanding has to be complemented by reliable tools that can in-silico predict the interaction and thus filtration efficiencies of the broad range of human viruses based only on their structure. In the following subsections, we discuss these four key topics that are relevant for virus adsorption, focusing on existing knowledge gaps and how to address them.

### Electrostatic Interactions

**Figure 2:**
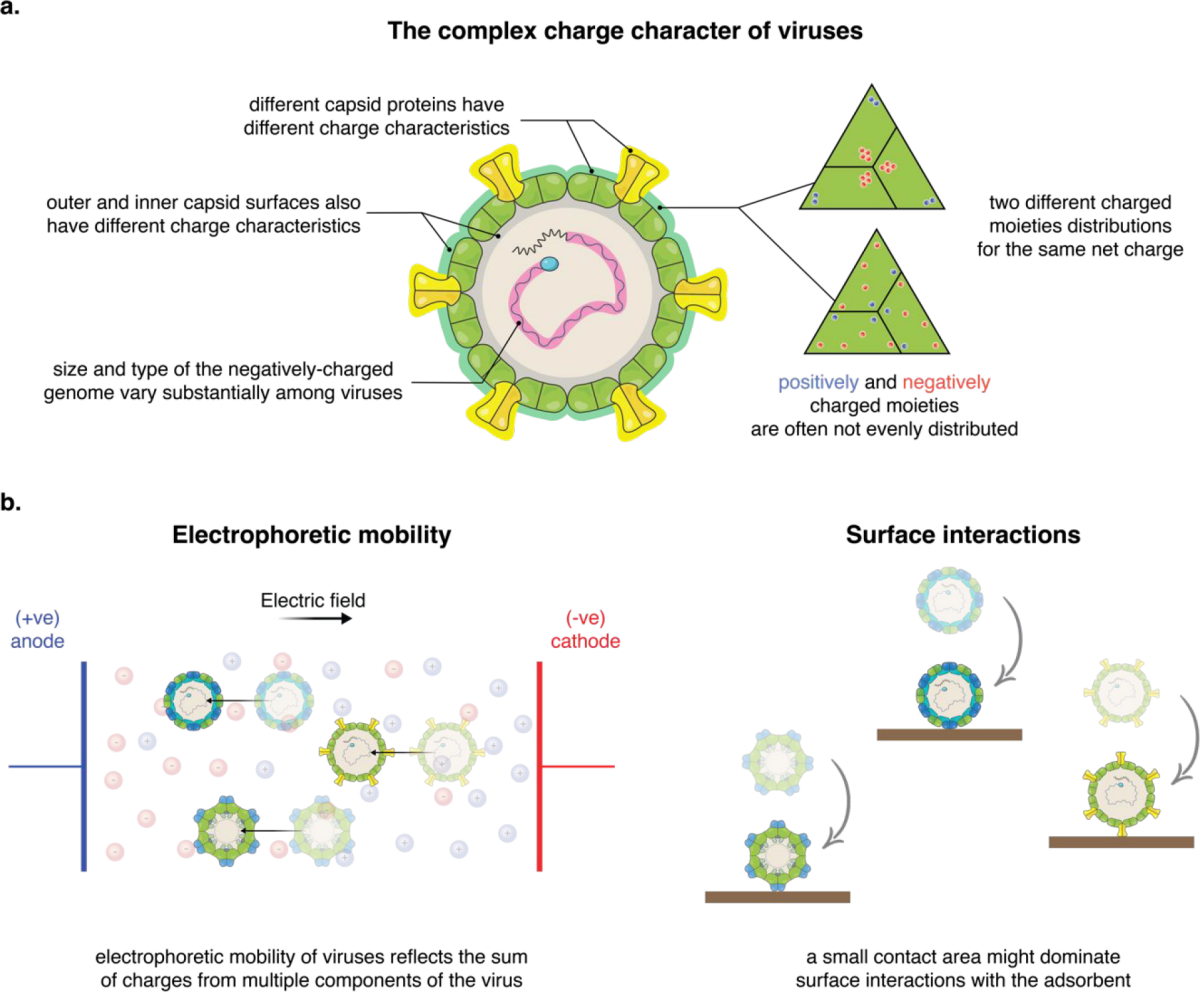
Electrostatic character and interactions of viruses. (a) Schematic depicting the complex charge character of viruses: all viruses contain a negatively charged genome, which can considerably vary in size and type; they are often composed of several capsid proteins which have different charge characteristics; their charged moieties are unevenly distributed on the inner and outer surfaces of the virus capsid; charges on the outer surface can have different spatial distributions. (b) Schematic showing the difference between the sensed charges by electrophoretic mobility-based methods and the ones that dominate surface interactions. While the electric field driving the electrophoretic mobility of viruses act upon several components simultaneously, at least on all charges on the outer surface of the virus, virus interactions at surfaces might be dominated by the charged moieties at a small contact area with the surface. Therefore, the charge character obtained from electrophoretic mobility might not reflect virus interactions, particularly for large viruses with heterogeneous surfaces and uneven morphologies.

Electrostatic interactions are often observed as a simplistic binary system: oppositely charged viruses and surfaces will attract and attach to each other, and similarly charged ones will repel and remain separated from each other. While the attraction of oppositely charged viruses and surfaces might hold correct for most cases, interactions of similarly charged ones exhibit more complex features. Some viruses can extensively adsorb even under electrostatically repulsive conditions, likely driven by attractive contributions from, e.g., the hydrophobic effect, cation bridging, and/or van der Waals interactions.^44,98−100^ In comparison to synthetic nanoparticles, which frequently exhibit uniform surface charge, viruses possess a rather complex charge character (Figure 2a). All viruses contain a negatively charged core composed of the virus genome, which can vary considerably in size and type, e.g., ssRNA, dsRNA, ssDNA, and dsDNA. Positively and negatively charged amino acid residues are unevenly distributed on the inner and outer surfaces of the virus capsid. Additionally, the surface morphology of viruses is very diverse, with some viruses having a quasi-smooth spherical surface and others having loops, knobs, and/or pillars protruding up to tens of nanometers; these features frequently have a different charge character to the rest of the virus capsid.^101^ A physicochemical descriptor of viruses that reflects their complex charge character is missing, impeding the development of tools that can quantitatively describe and predict electrostatic interactions of viruses.

The isoelectric point (IEP) of viruses is often used as a physicochemical descriptor to rationalize and speculate about the electrostatic interactions of viruses.^62,63,96^ The rationale is that, at pH values above the IEP, viruses will be negatively charged, thus adsorbing to positively charged surfaces and repelling from negatively charged surfaces; the other way around holds for pH values below the IEP. The IEP is, however, a single-value parameter and does not reflect the charge density of viruses at different pH values; viruses with very close IEPs can still exhibit a large variation in their charge density at pH values below or above the IEP.^44,102^ It is, therefore, necessary to use a descriptor that can distinguish virus charges at different pH values. Experimentally, this could be estimated based on the electrophoretic mobility of viruses. A variety of experimental approaches exist that can be used to determine the electrophoretic mobility of viruses. Approaches that can simultaneously distinguish biological macromolecules based on both their charge and size, e.g., capillary electrophoresis^103^ and tunable resistive pulse sensing (TRPS),^104^ are advantageous over methods that distinguish based on charge only. The results of the latter are often compromised by impurities in virus solutions.^105,106^ But even when using the proper experimental approach, there are still two key challenges that hinder the use of experimentally determined charges. The electric field that drives the electrophoretic mobility of viruses acts upon the whole virus and thus reflects the sum of charges from different components of the virus (Figure 2b). Conversely, virus interactions at interfaces usually occur at a contact area that is much smaller than the sum of components dragged by the electric field (Figure 2b). Therefore, a discrepancy between the measured charge and virus interactions could arise, particularly when assessing viruses with spatially heterogeneous charge distributions and uneven morphologies. Finally, experimental charge determination remains, at best, highly challenging for many human viruses.

For these reasons, several attempts have been made to find a calculable descriptor for the charge of viruses. Inspired by globular proteins, initial attempts used the sequence of capsid proteins to calculate the charge of ionizable amino acids at different pH values.^44,61,80^ This approach, however, showed large discrepancies compared to experimentally determined charges and to virus surface interactions.^44,62,63,80^ For example, based on such calculations, the bacteriophage MS2 is expected to carry a net positive charge at pH 6; however, at the same pH value, it shows strong repulsion from negatively charged surfaces, strong adsorption to positively charged surfaces, and electrophoretic mobilities indicative of net negative charge.^44^

Two main theories emerged to explain this inconsistency. The first suggests that there are contributions from the viral genome toward the net charge of viruses.^102,105,107^ This theory is experimentally supported by observations of different electrophoretic mobility between virus and virus-like particles (VLPs) without a genome.^105^ These results were, however, not consistent across literature, and some studies have also shown that VLPs had very similar adsorption behavior to that of genome-containing viruses.^44,108^ Despite the growing evidence that the genome contribution toward electrostatic interactions might be limited or negligible,^62^ whether this is also the case for viruses of different genome sizes and types remains to be confirmed. This is apropos because most of the aforementioned studies were conducted using small ssRNA viruses from the *Leviviridae* family.

A second, more plausible, theory is that the charge characteristic of viruses is dominated by the ionizable amino acids on the outer surface of the capsid, with negligible contributions from the buried amino acids, the ones on the inner side of the capsid, and the genome. Calculating viral charge based on the amino acids of the outer capsid showed a very promising correlation with electrostatic virus interactions at interfaces.^44,80^ The use of this approach requires knowledge of the 3D structure of the virus, as well as a method to identify the amino acid residues that reside on the outer surface of the capsid. Due to advances in structural biology techniques, the number of high-resolution 3D structures of viruses is increasing exponentially.^109^ Surprisingly, identifying amino acid residues on the outer surface of the capsid turn out to be the main challenge toward applying this approach; computational sorting of amino acid residues using tools, such as CapsidMap,^110^ does not agree with manual sorting using protein visualization softwares.^44^ This inconsistency calls for developing and verifying new computational approaches that can rapidly identify the position of amino acid residues for the hundreds of available virus structures without the need for tedious manual work. To circumvent the need for the 3D structures and identifying the position of amino acids, Heffron and Mayer^63^ suggested using the capsid protein sequence after excluding the known and predicted genome binding regions. While considering the 3D structure of viruses is inevitable, if we aim at obtaining a refined descriptor of virus charge, the results of Heffron and Mayer highlighted a potential bias in previous literature, which mainly relied on *Leviviridae* viruses.^62,63^ Unlike many other viruses, the genome of *Leviviridae* viruses binds extensively to the inner surface of the capsid protein, which could have contributed to the success of using the charge from the outer surface of the capsids in previous studies.^44,80^ For proper development of a charge descriptor, future investigations need to consider a broader range of viruses.

The above-mentioned computational approaches have considered the effect of neither the spatial distribution of charges nor the surface morphology of viruses. Positively and negatively charged moieties can have different spatial distributions on virus surfaces, e.g., clustered or spread out. Such variation in distribution could occur in both single-protein capsids and multiprotein capsids. To the best of the authors’ knowledge, the effect of the spatial distribution of the charge has not yet been investigated. In addition, viruses featuring uneven surfaces, such as Adenoviruses, Rotaviruses, and Astroviruses, present another challenge concerning how much these features contribute to surface interactions compared to the rest of the capsid proteins. Such relative contributions are also expected to vary with the thickness of the electrical double layer, which itself depends on the water chemistry.

Taken together, in order to make significant advances in our understanding and prediction of electrostatic virus interactions, it is necessary to develop and validate a calculable physicochemical descriptor for the charge of viruses. This descriptor has to reflect the 3D distribution of the charged moieties, taking into account their radial position, spatial distribution, and virus morphology. Achieving this end goal is practically bound to engineering bacteriophages for which each of the discussed parameters, e.g., spatial distribution of charge and virus morphology, can be varied systematically while maintaining the other parameters unchanged.

### The Hydrophobic Effect

The hydrophobic effect contributes significantly to virus adsorption at interfaces, resulting in virus adsorption even under electrostatic repulsion between negatively charged viruses and surfaces.^44^ Developing a quantitative framework/expression to describe the contribution of the hydrophobic effect to virus adsorption is, however, more challenging than in the case of electrostatics. Electrostatic interactions are theoretically well-explained with expressions accurately describing the energy and forces of the electrostatic double layer for different geometries.^111^ Such quantitative theoretical description of the hydrophobic effect is still missing; the available theoretical formulations lack the broad consensus in the scientific community. Research over the last few decades, however, is slowly revealing the physical origins of the hydrophobic effect.^112−121^ A quantitative description of the distance dependence of the hydrophobic effect has largely been impeded by experimental challenges;^120^ e.g., reports about the effective range of the hydrophobic effect varied between approximately ten nanometers^118^ and a few micrometers.^122^ Currently, it is thought that the decay length of these interactions is in the range of ≤2 nm and its effective range is up to a few tens of nanometers.^120^ Equations describing the distance-dependent hydrophobic interaction potentials have been suggested for interacting planar surfaces and nanoparticles.^123−125^ These advances will hopefully lead to a unifying theoretical formulation to describe the hydrophobic effect. However, it is crucial to point out that, like electrostatic interactions, these formulations are based on homogeneous, smooth surfaces and nanoparticles. Therefore, in addition to the lack of a unifying theoretical formulation, the heterogeneous surface chemistry and morphology of viruses present another hurdle, as discussed for electrostatic interactions. These two challenges have several implications for assessing the contributions from the hydrophobic effect toward virus interactions, which will be discussed hereafter.

Both experimental and computational attempts to quantify the hydrophobic character of viruses yield an empirical qualitative ranking, rather than a quantitative descriptor. Experimental methods available to study hydrophobicity include microbial adhesion to hydrocarbons (MATH), hydrophobic interactions chromatography, reverse-phase chromatography, ANS fluorescence, and aqueous two-phase systems (ATPS).^126^ With very rare exceptions,^127^ their application to study viruses is almost nonexistent, likely due to several experimental challenges. This led to the development of new experimental approaches that are more suitable for ranking viruses according to their hydrophobic character, e.g., adsorption to hydrophobic surfaces^44,89^ and SDS-modified capillary zone electrophoresis (CZE).^103^ Adsorption to hydrophobic surfaces also includes electrostatic contributions, because hydrophobic surfaces acquire a pH-dependent negative charge in aqueous environments,^128−130^ thus rendering ambiguous ranking that reflects both the hydrophobic and electrostatic character of the viruses. SDS-modified CZE relies on the observation that the electrophoretic mobility of viruses shifts toward more negative mobility in the presence of SDS,^131^ which is attributed to binding of the SDS to hydrophobic patches on the surface of the viruses exposing its negatively charged polar end, thus increasing the negative charge of the viruses. While offering a promising experimental tool to elucidate virus hydrophobicity, further assessment and validation might be needed before being widely accepted by the research community. In particular, the potential bias if SDS binds with its negatively charged end to the positively charged moieties on the surface of the virus, rendering them neutral; this would erroneously attribute viruses with higher positive-charge densities as more hydrophobic.

Even when using such novel approaches, the challenges discussed with experimental determination of virus charge also apply to hydrophobicity: (i) measured values reflect the hydrophobicity of the whole virus, while virus adsorption to surfaces might be dominated by single components of the virus or a small patch on the virus surface; and (ii) experimental results are unattainable for many human viruses. Three computational approaches have been proposed to assess the hydrophobic character of viruses, the earliest of which assigns different scoring indices for each amino acid residue^132−134^ and calculates a moving average based on the sequence of the capsid proteins.^44,80,103^ Such calculations can be readily performed using the ProtScale application on the Expasy server.^135,136^ This approach yields what is known as the hydropathy index plots. These plots, however, offer no quantitative measure of the hydrophobicity of viruses, thus hindering direct and quantitative comparisons of different viruses. Another approach relies on calculating the ratio of apolar (hydrophobic) solvent accessible surface area (SASA) to the total SASA of the virus capsid. When applied to four different viruses of the *Leviviridae* family, this approach yielded almost identical values for the four viruses, despite the clear difference in their surface interaction with hydrophobic surfaces.^44^ Moreover, no improvement was observed when this approach was applied only to the amino acids on the outer side of the capsids.^44^ A more intricate approach was achieved by applying a scoring system that exponentially increases with the area of each apolar patch on the outer surface of the virus capsid,^137^ in which the total hydrophobic score is equivalent to the sum of the scores of all hydrophobic patches on the outer surface of the capsid. When applied to the same four viruses, the calculated score could very well explain the hydrophobic interactions observed in virus adsorption experiments.^44^ Further verification of this approach is still needed, ideally using engineered bacteriophages that have the same amino acid residues on their surfaces but distributed differently to form hydrophobic patches of different sizes. Additionally, a representative physicochemical descriptor for the hydrophobic character of viruses would also need to reflect the surface morphology of the viruses.

### Water Chemistry

The effect of water chemistry, i.e., pH, ionic strength, and ionic composition, on electrostatic interactions is well documented. The change in pH alters the protonation state of ionizable moieties, thus changing the charge of both viruses and adsorbing surfaces; e.g., as pH decreases, more moieties are protonated, thus decreasing the net negative charge of a virus and/or a surface, provided that both are above their IEP.^44,84^ Changes in virus and surface charges with pH have a substantial effect on virus adsorption to interfaces;^44,70,81,87,138,139^ for example, while MS2 showed no detectable adsorption at pH > 6 to a negatively charged surface composed of carboxyl-terminated self-assembled monolayers, it extensively adsorbed to the same surface at pH 5.^44^ Ionic strength also has a well-elucidated effect on electrostatics; the increase in ionic strength screens the electric double layer and thus shields electrostatics. This is particularly manifested in the case of electrostatic repulsion, in which the increase in ionic strength results in shielding the repulsive forces, allowing viruses to attach to the surface via interactions, such as the hydrophobic effect.^44,84,138^ Changes in ionic composition also influence virus adsorption; arguably, the most striking change in virus interactions occurs when multivalent cations are added or removed from solution.^76,78,90,98,140−143^ For instance, the addition of 1 mM Ca^2+^ increased the adsorption efficiency of MS2 to NOM by almost 2 orders of magnitude.^140^ Multivalent cations are orders of magnitude more efficient than monovalent ones in reducing the electric potential at interfaces; e.g., a CaCl_2_ electrolyte will be approximately 100 times more effective in reducing the potential than a NaCl electrolyte with the same molar concentration.^111^ Not only do multivalent cations affect the electric potential of charged entities, but they can also bind to negatively charged moieties, thus altering the surface charge and reducing the (negative) electric potential further.^111^ This binding can mediate what is known as cation bridging, in which a cation bridges two negatively charged moieties, e.g., two carboxyl groups on the surface of a virus and an adsorbent. It has been speculated that cation bridging occurs only with specific negatively charged moieties, particularly carboxyl groups.^84^ For waterborne viruses, Mg^2+^ and Ca^2+^ are the two most relevant multivalent cations due to their abundance in surface water.^144^ Initial reports suggested that Ca^2+^ is more efficient than Mg^2+^ for cation bridging.^140^ Using locally high concentrations of divalent cations at the interface between the filtration material and water is a potential, but unexplored, approach to enhance adsorption-based filtration of viruses. However, certain knowledge gaps about the role of divalent cations need to be addressed first. (i) The specificity of cation bridging to carboxyl groups needs to be verified. (ii) If cation bridging is specific to carboxyl groups, does this imply that the higher the density of these groups, the larger the capacity of virus adsorption, or would electrostatic repulsion override the effect of the cations? (iii) What happens to the viruses under depletion conditions of the divalent cations? Do they desorb or remain attached, and how is this affected by the physicochemical properties of the adsorbent? (iv) Is Ca^2+^ indeed more efficient than Mg^2+^? If yes, in which molarity range is this difference noticeable?

It is important to mention that solution chemistry affects not only electrostatic interactions but also the hydrophobic effect. Hydrophobic surfaces tend to acquire a net negative charge in aqueous environments. This charge depends on pH, ionic strength, and ionic composition, i.e., multivalent cations,^128−130^ which is also reflected in virus adsorption to hydrophobic surfaces.^44^ This is worth noting because, taking into consideration the very diverse scientific community that studies virus adsorption, this phenomenon might go unnoticed.

### Effect of Coadsorbates

**Figure 3:**
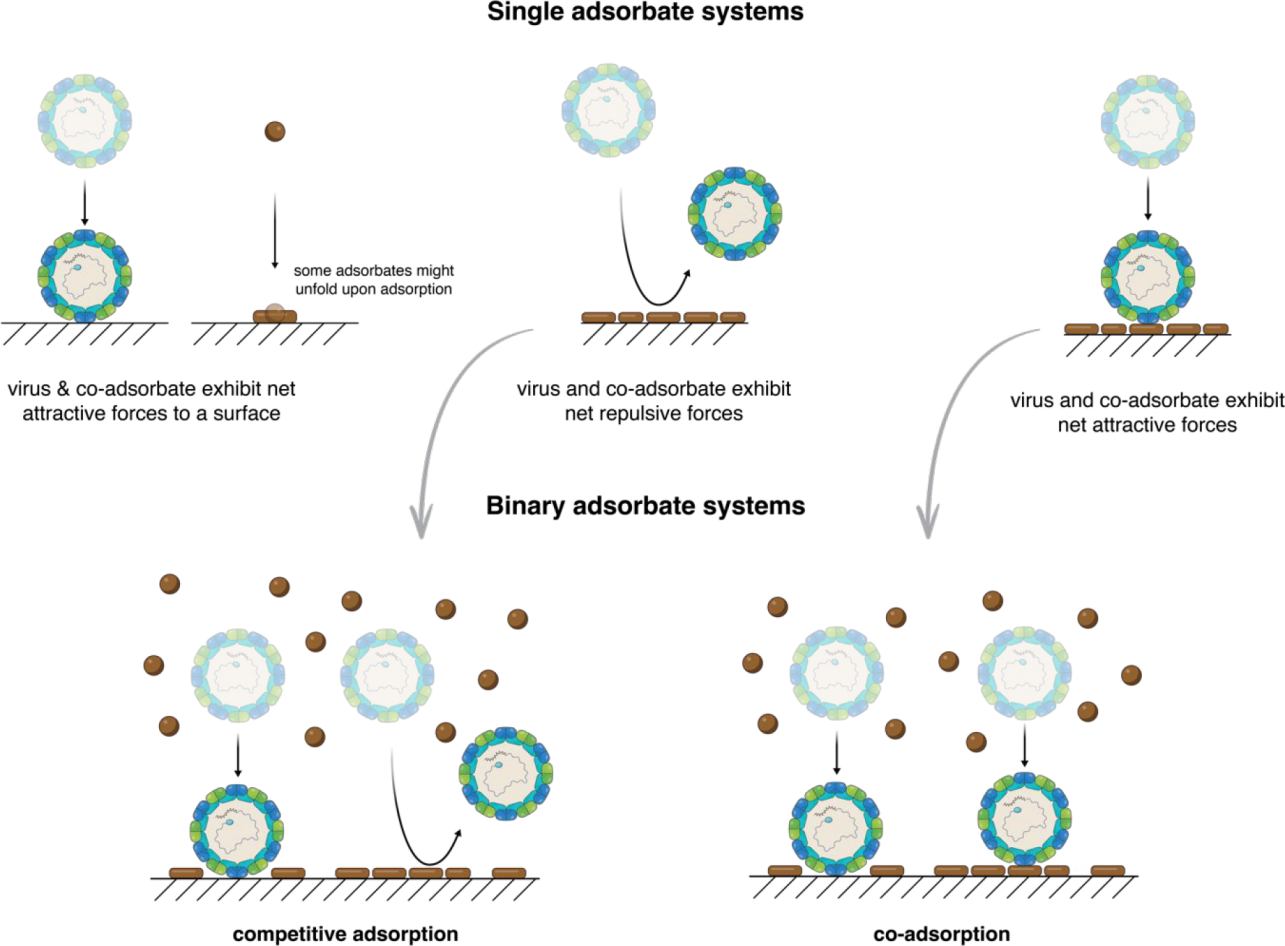
Competitive and coadsorption systems. Schematics showing two different scenarios for adsorption of virus in the presence of a coadsorbate. If the virus and the coadsorbate have net repulsive interactions, then they will experience competitive adsorption and thus a significant decrease in the adsorption capacity for the virus. Conversely, if they have net attractive interactions then they will coadsorb with limited effect on the adsorption capacity for the virus. Adapted from ref (45). Copyright 2016 American Chemical Society.

In our discussion of the effect of coadsorbates on virus adsorption, we mainly consider NOM, which is arguably the most abundant and ubiquitous coadsorbate in water and wastewater.^145−148^ NOM possesses a net negative charge at circumneutral pH values,^149^ thus likely exhibiting comparable electrostatic interactions to many viruses. Several studies have demonstrated that the presence of NOM suppresses virus adsorption.^70,78,79,86,138,150−153^ This effect was, however, virus-dependent: for example, while MS2 adsorption to soil minerals and sand was suppressed in the presence of NOM, little to no effect was observed in the case of ΦX174.^151^ The mechanism by which coadsorbates affect virus adsorption was neatly demonstrated using in situ adsorption experiments to positively charged surfaces of four different viruses that exhibit different affinities to NOM.^45^ The results showed that viruses, e.g., MS2, that show net repulsive interactions with NOM exhibit markedly reduced adsorption in the presence of NOM, whereas, viruses, e.g., Qβ, that show net attractive interactions with NOM are only minimally affected by the presence of the NOM (Figure 3).^45^ As anticipated, the suppressive effect of NOM on adsorption of some viruses was dependent on NOM concentration: the higher the NOM concentration, the fewer viruses that were adsorbed. Using a random sequential adsorption (RSA) model for a binary system of adsorbates, the study also revealed the significant effect of minor interaction details on the adsorption capacity of viruses, e.g., the minimum contact area required for virus attachment to the surface and the unfolding, i.e., spreading out, of NOM upon adsorption to surfaces.

The suppressive effect of NOM on virus adsorption presents a massive challenge for adsorption-based filtration. Novel and innovative ideas to overcome such challenge will likely emerge if a deeper understanding is obtained of multi-adsorbate systems in complex environments. For example, little is known about how controlling the size and pattern of attractive patches on a surface would affect the adsorption kinetics and capacity of viruses in such systems.

## Virus Inactivation at Interfaces

Virus adsorption to certain surfaces, such as iron (oxy)hydroxides, has been shown to accelerate virus inactivation.^79,99,100,154,155^ This is often mediated by chemical reactions with reactive oxygen species (ROS) that are present with locally high concentrations in very close proximity to such surfaces and rapidly decay even at short distances from the surface.^99,155^ However, the production of ROS often requires sunlight or the addition of chemicals, such as H_2_O_2_.^99,155−158^ The need for light exposure or the addition of chemicals complicates the use of adsorption-based filters and runs counter to the original objective of developing facile technologies that can be used for POU water treatment. Some research results have indicated that strong interactions between viruses and surfaces could potentially result in virus disintegration and thus inactivation.^79,100,154^ Based on experiments with isotopically labeled viruses, Murray and Laband^100^ postulated that van der Waals interactions between Poliovirus and CuO could result in virus disintegration. Using a similar approach, Ryan et al.^154^ suggested that strong electrostatic interactions of PRD1 and MS2 with iron oxides can cause virus disintegration. Gutierrez et al.^79^ recovered only 2% of infection Rotavirus after interaction with Hematite; in comparison, 64% of infectious MS2 was recovered. TEM images suggested that Rotavirus might have been damaged by its interactions with Hematite. Conversely, in situ adsorption experiments of MS2, fr, GA, and Qβ to positively charged surfaces, under conditions favoring very strong attractive interactions, have shown no signs of viral disintegration.^44^ Unequivocal evidence of virus disintegration due to physical interactions only is still missing, as well as the mechanism behind such disintegration. Indeed, the search for a surface that can spontaneously inactivate nonenveloped viruses should be unrelentingly pursued. Future research should also investigate the potential synergetic effect of various parameters, such as surface roughness, charge, and hydrophobicity, on virus inactivation. Identification of surface properties that have virucidal effects would constitute a seminal advancement in our fight against waterborne viruses.

## Experimental Considerations

The scientific community that is interested in virus adsorption to solid–water interfaces is a very broad one, including, among others, material scientists, environmental chemists, microbiologists, food scientists, virologists, and physicists. In this section, we briefly discuss a few experimental considerations to assist in avoiding misinterpretation of experimental results.

### Virus Solution Purity

High purity virus solutions are a prerequisite for successful physicochemical characterization of viruses and for mechanistic adsorption studies. It has been previously shown that different purification protocols result in different electrophoretic mobilities,^105,106^ size distributions,^105,106^ and adsorption behavior^44^ of viruses. The effect of impurities on virus adsorption is mechanistically similar to the effect of coadsorbates depicted in Figure 3. Virus adsorption studies are often conducted by either measuring the decrease in virus concentration after adsorption or by monitoring mass change using in situ surface-based techniques. For the former, impurities could result in a substantial underestimation of the adsorption capacity. For the latter, and since mass monitoring is not specific to viruses, impurities could additionally result in erroneously reporting adsorption of impurities as adsorption of viruses. If not intentionally inspected, the impact of impurities can, in many cases, go unnoticed.^44^

Various protocols exist for virus propagation, purification, and concentration, the most used of which are PEG precipitation, CsCl density gradient centrifugation, and buffer washing using dialysis or centrifugal ultrafiltration. Some protocols also comprise a combination of these methods.^46^ While some studies claim that using the CsCl gradient yields the highest purity,^105,106^ there are not enough studies to reach a generic recommendation. Variations in the rigor of experimental practice could also compromise the purity of the virus solution, even when using the same protocol. Furthermore, it has been reported that viruses might disintegrate over time, even when stored in the dark at 4 °C, thus interfering with mechanistic adsorptions studies.^44^ Irrespective of the purification protocol, it is, therefore, recommended to periodically run purity control experiments, e.g., adsorption experiments under conditions of well-known theoretical and experimental results.^44^

### Quantification of Viruses and Their Integrity

**Figure 4:**
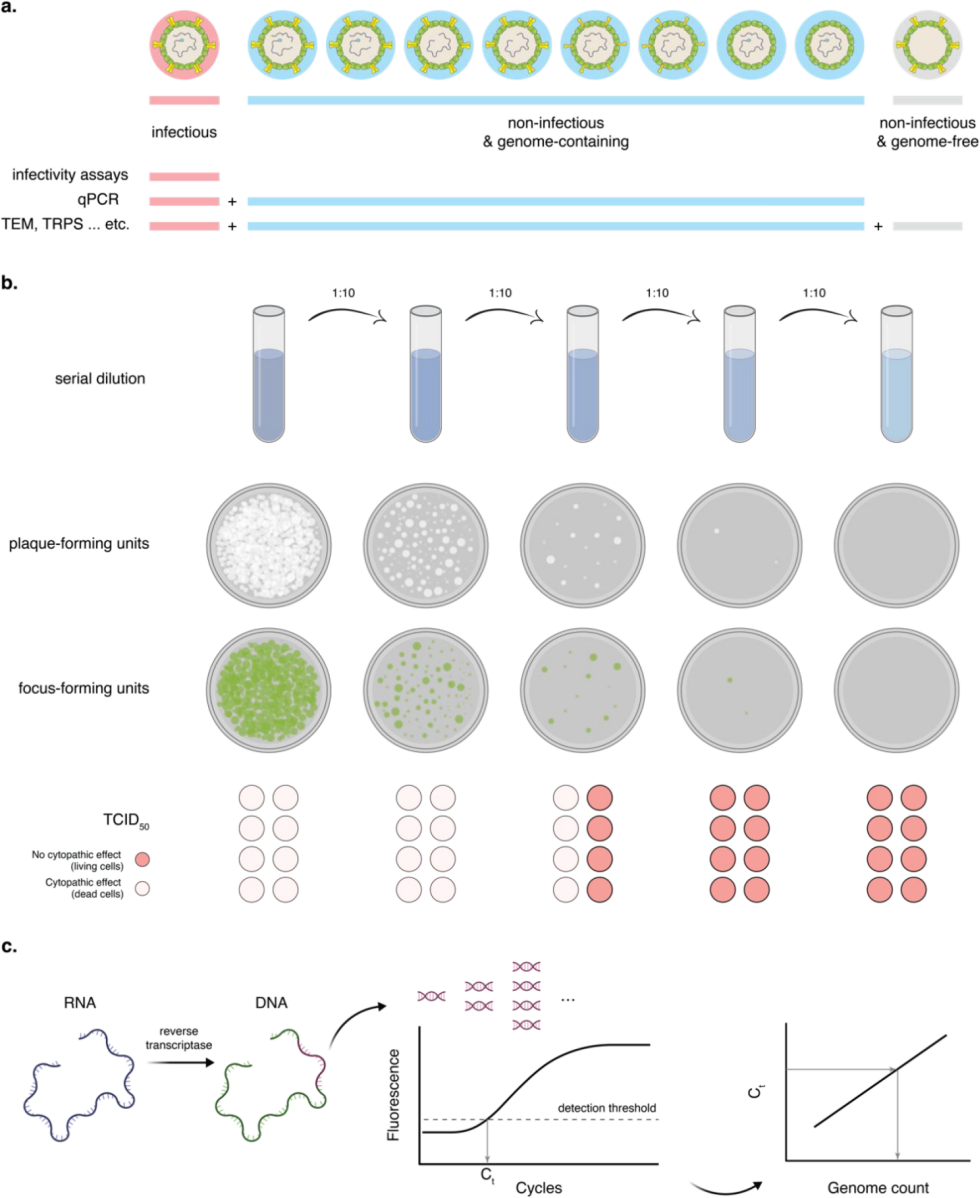
Quantification of viruses. (a) Schematic showing the different populations that exist in a virus solution: infectious viruses, noninfectious/genome-containing, and noninfectious/genome-free. Different quantification methods, e.g., infectivity assays, qPCR, and TEM, will assess different populations. (b) Schematics showing three different ways to express the concentration of infectious viruses: plaque-forming units, focus-forming units, and TCID_50_. (c) Schematic showing RT-qPCR to determine the genome count of viruses. The RT, i.e., reverse transcriptase, step is only needed for RNA viruses. Viruses containing incomplete genomes that have the segment being replicated in the PCR will still be counted: the contrary also applies; viruses containing incomplete genomes that do not have the segment being replicated will not be counted.

Virus solutions contain several populations: infectious viruses, noninfectious/genome-containing viruses, and noninfectious/genome-free viruses (Figure 4a). The two most common ways to determine virus concentrations are quantifying the number of infectious viruses and the number of genomes, i.e., genome count (Figure 4b,c). The number of infectious viruses is usually determined using plaques assays or median tissue culture infectious dose (TCID_50_). In the plaque assays, the virus-containing solution is plated with the host cells in a Petri-dish; after the due incubation period under optimized conditions for cell growth, the number of plaques, i.e., spots where cells were lysed by infectious viruses, is counted. Each plaque represents one infectious virus, and the virus concentration is expressed as plaque forming units (PFU)/ml. A special variation of plaque assays is the focus forming assay (FFA), which is commonly used for viruses that do not lyse the host cells and thus do not form plaques. FFA utilizes an immunostaining technique using fluorescently labeled antibodies that are specific to one of the viral proteins. Infected cells will exhibit a high virus concentration in their vicinity, forming clusters (foci). Virus concentrations are thus represented in focus forming units (FFU)/ml. TCID_50_ is an end point assay in which the end point reflects the dilution that results in killing 50% of the hosts. TCID_50_ is assessed by either monitoring the death of the host, e.g., laboratory rats, or monitoring the cytopathic effect of host cells; it is represented as TCID_50_/ml. Both plaque assays and TCID_50_ are performed for a dilution series of the solution under consideration. The large majority of virus filtration/inactivation studies report virus infectivity only, either in PFU/ml or TCID_50_/ml, which is the most relevant parameter when assessing the efficiency of the filtration/inactivation approach and the safety to consume the effluent water. The efficiency of filtration is often reported as log reduction values (LRV) in the virus concentration of the effluent over the influent concentration, where 1 LRV is equivalent to 90% filtration efficiency, 2 LRV ≡ 99% filtration efficiency, 3 LRV ≡ 99.9% filtration efficiency, etc.

**Figure 5:**
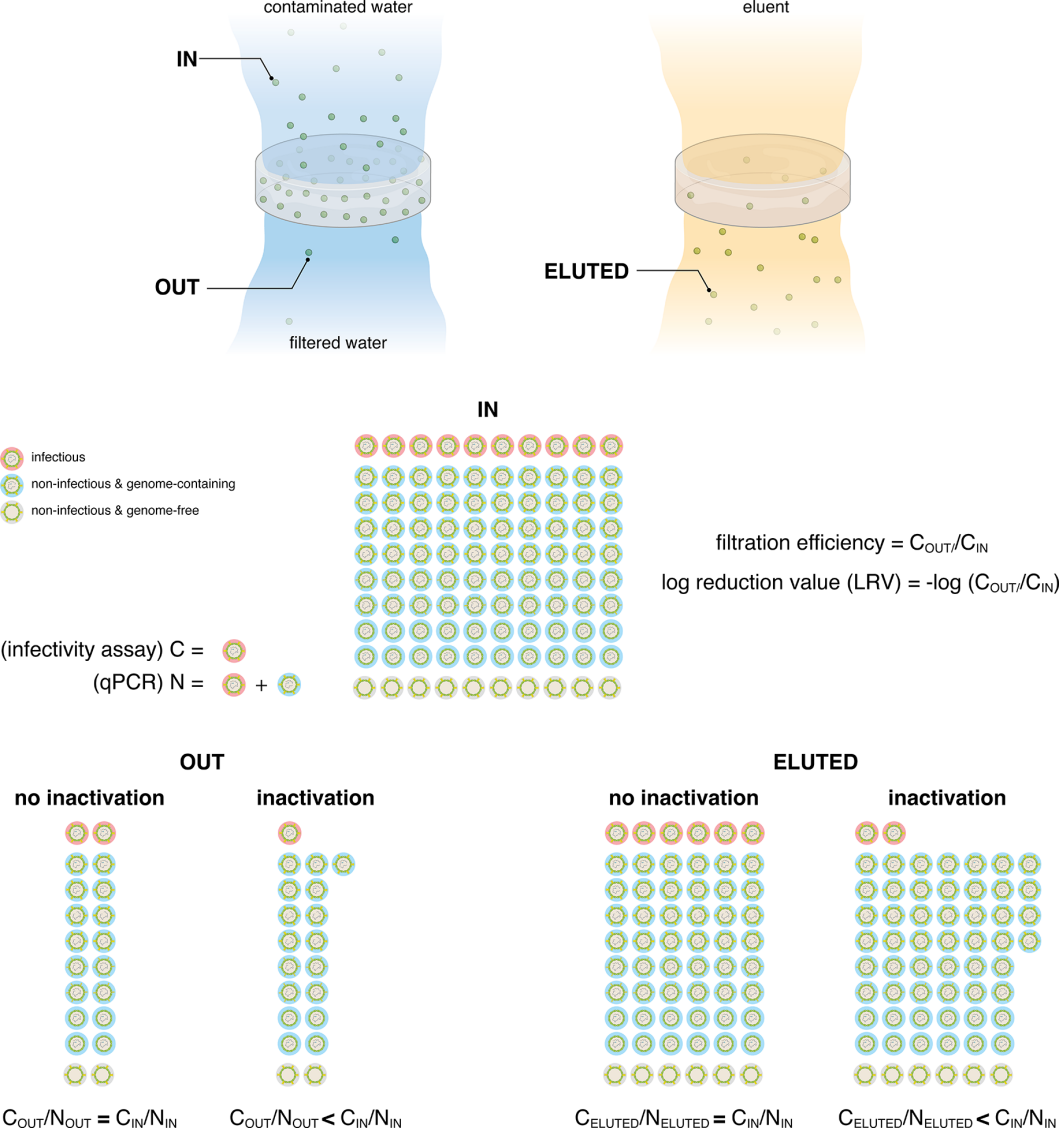
Adsorption versus adsorption and inactivation. Schematics showing how to determine filtration efficiency and differentiate between filters that adsorb viruses only or other ones that adsorb and inactivate viruses. IN: represents virus concentration of the contaminated water; OUT: virus concentration after filtration; ELUTED: virus concentration after eluting adsorbed virus using an eluent, usually a high pH buffer solution. These calculations were based on the following two assumptions: no preferential adsorption or elution of any of the virus populations; the inactivation mechanism does not cause damage to the genome segment that is replicated in the qPCR quantification.

However, by measuring infectivity alone, it is not possible to differentiate between inactivated and irreversibly adsorbed viruses, as both of them will result in a reduction in the concentration of infectious viruses in the effluent. Simultaneous determination of the genome count offers a better understanding of the filtration/inactivation process since it also enables determining the portion of viruses that has been inactivated. The genome count is determined using real-time polymerase chain reaction (qPCR), in which a segment of the viral genome is replicated through PCR cycles taking place in a thermocycler. The number of cycles required to achieve a detectable concentration of the genome is then used to calculate the genome count in the original sample based on a calibration curve. It is important to note that the genome count of lab-propagated viruses is often 1 to 3 orders of magnitude higher than the number of infectious viruses.^44,46^ As viruses get inactivated, the ratio of infectious viruses to the genome count decreases; this ratio is particularly important when evaluating the efficiency of virus inactivation by any approach. Viruses could get inactivated while passing through the filter, e.g., due to interaction with dissolved ions. They could also get inactivated after attaching to the filter media. In order to assess the inactivation of attached viruses, it would be necessary to first elute them and assess their infectivity and genome count. Elution efficiency varies based on the virus and the adsorbent.^99,155,159,160^ Assuming that there is no preferential adsorption or elution of infectious or noninfectious viruses, the decrease in the ratio of the infectious viruses to genome count in the effluent or the eluent is a strong indicator of virus inactivation (Figure 5).

While these are the most common approaches to quantity virus concentration, many other different techniques also exist, e.g., cryo-transmission electron microscopy (cryo-TEM) to count the number of virus particles, protein assays to determine the protein concentration of a virus, enzyme-linked immunosorbent assay (ELISA) to determine the concentration of an antibody-binding virus protein, and tunable resistive pulse sensing (TRPS) for counting virus particles. Each of these approaches measures a different aspect of the virus concentration.

## Selected Examples of Adsorption-Based Filters

**Figure 6:**
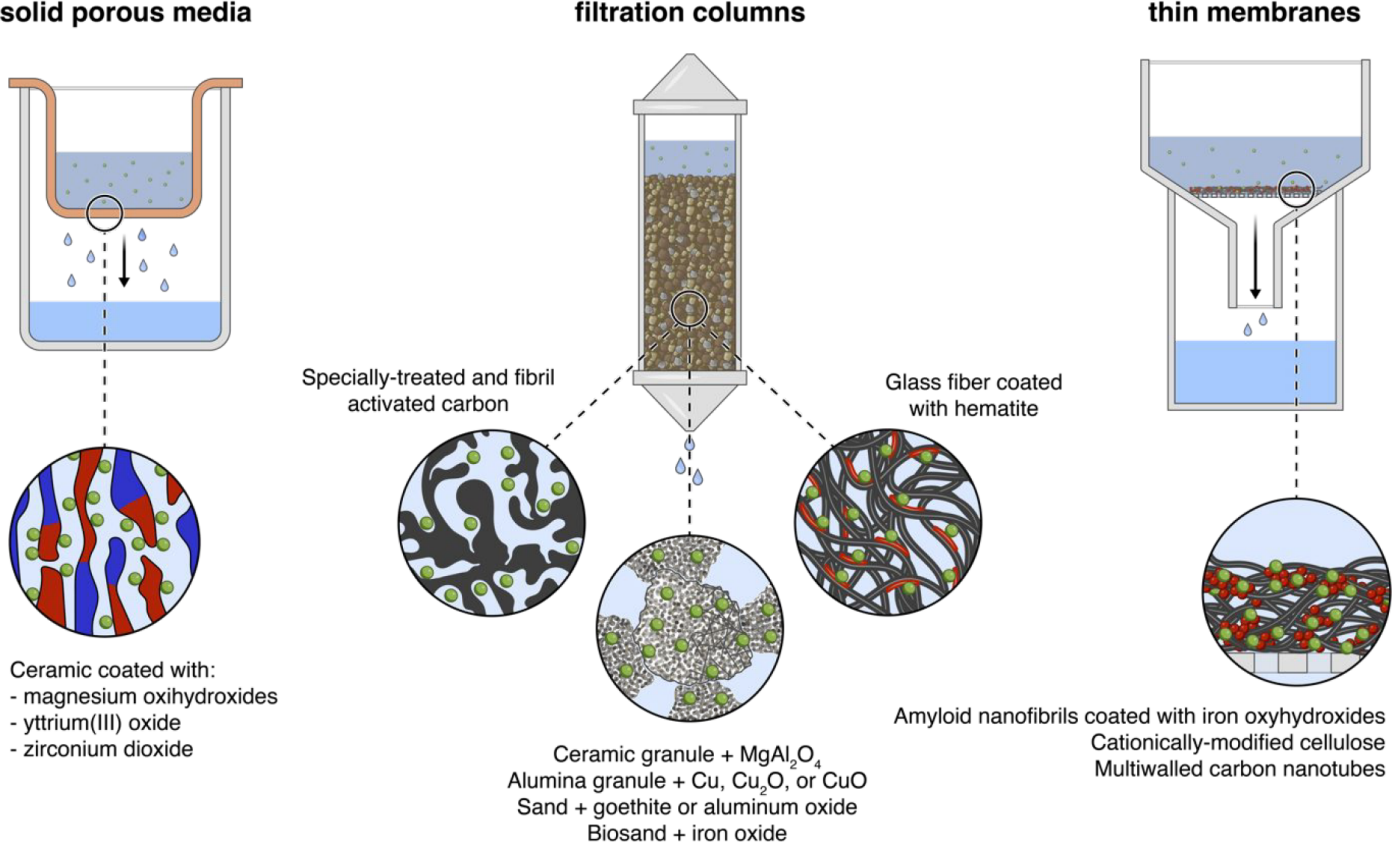
Selected examples of adsorption-based filters. Most adsorption-based filters come in one of the three depicted forms: solid porous media,^70,71,161^ filtration columns,^69,72,74,75,79,162−164^ and thin membranes.^46,73,138,139,165,166^ The schematics are artistic representations of the selected filters; they are not drawn to scale and should not be interpreted as an accurate depiction of the mentioned filters. Green circles represent viruses, and the red color represents the coating or nanoparticles that adsorb viruses.

Numerous filter materials have been tested to trap waterborne viruses.^40^ Most of these filters take one of three forms: solid porous media,^70,71,161^ filtration columns,^69,72,74,75,79,162−164^ and thin membranes^46,73,138,139,165,166^ (Figure 6). With very few exceptions, these materials are composed of a skeletal component and a coating/decoration. The former makes the bulk material and could be, among others, ceramics,^70,71,75,161^ alumina,^74^ multiwalled carbon nanotubes (MWCNTs),^138,139,165,166^ cellulose,^73^ glass fibers,^79^ or amyloid fibrils.^46^ The latter comprises the adsorption sites for virus attachment and is often made of metal oxide coating or nanoparticles, for example, magnesium oxyhydroxide,^71^ copper and copper oxides,^74,166^ and iron oxyhydroxide.^46^ In the following section, we will briefly discuss three recent innovations which achieved notable advances in the field; a comprehensive literature review of adsorption-based filters has been recently covered by Sellaoui et al.^40^

Canh et al.^72^ produced novel porous carbon (NPC) from rice husk by silica removal, followed by steam activation and acid rinse. The material could be considered as a special form of activated carbon (AC). Conventionally produced AC often shows low filtration efficacy of viruses but is particularly effective against small molecule contaminants. The results of Canh et al. showed a significant improvement over previous attempts with AC, reducing the incubation time from 8 h to 60 min while achieving approximately 5 LRV of MS2. The experiments were also unintentionally conducted in the presence of 6.7 mg/L T0C; while being a very low concentration, it shows that the NPC could still be effective under minor competitive adsorption conditions from dissolved carbon. Particularly important is that the material is produced from rice husk and thus is in full accordance with the global need for waste valorization.

Palika et al.^46^ produced a viral trap made of amyloid fibrils and iron oxyhydroxide nanoparticles. The material showed more than 5 LRV of MS2 and approximately 0.5 LRV of Enterovirus 71. The material seems to also have a partial inactivation effect on MS2; while the inactivation effect is not pronounced, it presents promise for further optimization to reach higher efficiencies of inactivation. The material offers one of the most environmentally friendly approaches to water filtration. Its main component is β-lactoglobulin (BLG), which is derived from byproducts of the dairy industry. The second component of the material is iron oxyhydroxides, which is a naturally existing material with no toxicity to humans or the environment. In addition, the production of the material requires very little energy and no use of harsh chemicals or petrochemicals. A closely related material, amyloid fibrils-carbon hybrid, has been previously used to filter a broad range of contaminants, including heavy metal ions, metal cyanides, and nuclear waste,^167^ opening the possibility for broader applications of a further developed version of the material virus filtration and beyond.

Yüzbasi et al.^75^ recently developed a granular material with a ceramic core coated with MgAl_2_0_4_. The material showed high efficiency in removal of MS2 and fr, in which only 4 g of the material was enough to achieve >7 LRV of 1 L of virus-contaminated water. The efficiency remained the same for fr, even when filtering 2 L; for MS2, the efficiency went down to approximately 4.5 LRV when filtering 2 L of virus-contaminated water. The granules rely on low-cost raw materials and are chemically and mechanically very stable. Their high stability enables regeneration of the material by backing at 400 °C. While this is a relatively high temperature and the real extent of regeneration needs to be further assessed, the possibility to regenerate virus filters by applying heat only, without the need for chemical cleaning, is a major step toward sustainable filtration solutions.

## Outlook and Conclusions

Adsorption-based filtration offers a propitious approach to address the challenge of providing safe drinking water for an enormous world population of almost 8 billion people, with minimal, if any, negative impact on the climate crisis and the environment. Despite the progress achieved through years of research and assessment of different materials, a filter that can meet all the expectations is still missing. We argue that achieving substantial progress toward developing such filters would be built on a better fundamental understanding of virus interactions and inactivation at solid–water interfaces. Achieving such an understanding entails adopting new descriptors for the physicochemical descriptors of viruses, advancing beyond traditionally used descriptors that were mainly developed to describe homogeneous, smooth, and spherical nanoparticles. Viruses are, in contrast, chemically heterogeneous with very diverse morphologies. To this end, it is necessary to harness the power of newly developed microbiological tools, in particular the versatility offered by advances in targeted gene engineering of bacteriophages. Additionally, the power of computational tools has to be exploited in order to use such physicochemical descriptors to predict the efficacy of newly developed filters for the broad variety of human waterborne viruses. Ultimately, top-down and bottom-up approaches from the molecular to the colloidal scale will have to be successfully combined to meet all the challenges associated with the design of efficient waterborne virus traps.
